# Supplementary material for: Provider Preference, Logistical Challenges, or Vaccine Hesitancy? Analyzing Parental Decision-Making in School Vaccination Programs: A Qualitative Study in Sydney, Australia
Source: Vaccines (Basel). 2025 Jan 17;13(1):83. doi: 10.3390/vaccines13010083 (PMC11768876; doi:10.3390/vaccines13010083)
Supplement: Supplementary file 1 [file vaccines-13-00083-s001.zip › Table S1.pdf]

SUPPLEMENTARY MATERIALS

Table S1: Characteristics of school staff

School Staff Code	Sex	Co-education	School Sector	Consent process	Substantive Role in School
S1	F	**Combined**	Government	Paper	Teaching
S2	F	**Combined**	Government	Paper	Teaching
S3	F	**Combined**	Government	Paper	Teaching
S4	M	**Combined**	Catholic	Online (prior to first clinic)	Executive
S5	F	**Combined**	Independent Christian	Online (prior to first clinic)	Administrative
S6	M	**Combined**	Government	Online	Teaching
S7	F	**Combined**	Independent Anglican	Online	Administrative
S8	F	**Girls**	Catholic	Online	Administrative
S9	F	**Girls**	Government	Online	Executive
S10	F	**Combined**	Government	Online	Teaching
S11	F	**Combined**	Government	Online	Teaching
