# Supplementary material for: Provider Preference, Logistical Challenges, or Vaccine Hesitancy? Analyzing Parental Decision-Making in School Vaccination Programs: A Qualitative Study in Sydney, Australia
Source: Vaccines (Basel). 2025 Jan 17;13(1):83. doi: 10.3390/vaccines13010083 (PMC11768876; doi:10.3390/vaccines13010083)
Supplement: Supplementary file 1 [file vaccines-13-00083-s001.zip › Table S2.pdf]

Table S2: Characteristics of parent participants

Parent Code^	Parent education	Child's gender	School Type	Received ≥1 school vaccination	Child's vaccination status	Method
P1	Certificate III	Female	Government	No	Unvaccinated	
P2	University (postgraduate)	Male	Independent	No	Unvaccinated	
P3	University	Female	Independent	No	Fully vaccinated at GP	
P4	University	Male	Government	No	Fully vaccinated at GP	
P5	University	Female	Government	Yes	Partially vaccinated (dTpa only)	
P6	University	Male	Government	No	Unvaccinated	
P7	Diploma	Male	Government	No	Unvaccinated	
